# Primary structure of proteins as a nanowire for metabolic electronic transport

**DOI:** 10.1186/s11671-015-0763-0

**Published:** 2015-03-12

**Authors:** Anatol D Suprun, Liudmyla V Shmeleva

**Affiliations:** Departments of Theoretical Physics, Faculty of Physics, Taras Shevchenko National University of Kyiv, Volodymyrska Street, 64/13, Kyiv, 01601 Ukraine

**Keywords:** Protein macromolecule, Nitrogen-oxygen model, External field, Residual field, Numbers of filling

## Abstract

It is considered that the major process in an organism is the synthesis of the adenosine triphosphate (ATP) molecules (its resumption from the adenosine diphosphate (ADP) molecules). These molecules are the basic (if not unique) energy resource of an organism. For the completion of process of the ATP synthesis in mitochondria, it is necessary to transfer to it a pair of electrons from places where electrons rise up as a result of oxidizing processes. Research of mechanisms of such transfer is important therefore, in particular, from the point of regulative influence on them in medical aims.

Various proteins, the primary structure of which can provide the transport of electrons between donors and acceptors, saturate a volume and membranes of cages. A question about a possibility to examine this primary structure of proteins as a nanowire of a semiconductor nature is analyzed. The possibility of active transport of electrons through its conductivity band is analyzed also.

In this paper, it was shown that a heterogeneous protein system is possible to be considered as a semiconductor with an average-nitrogen nuclear subsystem and with an average-oxygen electronic subsystem. Also, it was shown that in the potential energy of interaction between the electron and the nuclear subsystem indeed exists non-compensated contributions. These contributions are related to the radicals and provide the active transport of electrons along the primary structure of protein molecules.

It was demonstrated also that external fields can have local regulative influence on the transport of electron in proteins by compensating the remaining field or strengthening it.

Fulfilled analysis gives a possibility in zero approximation of the application of representation of numbers of filling to the protein molecule, considering it as the nanowire.

## Background

The detailed experimental and theoretical investigations showed that the energy structure of protein molecules in relation to the electronic states looks like enough typical for semiconductor crystals [1-10]. The first discussions of this question rose up, presumably, more than 70 years ago [1,2]. Already then, supposition about the semiconductor nature of such conductivity spoke out. Later, it was set that the injection of an electron from a donor (electronic conductivity) was carried out from the N-end of a protein molecule [3] and recently was set [4] that the withdrawal of the electron by an acceptor (hole conductivity) was carried out with its C-end. The first quantum calculations of the single-electron energy states of protein-similar polymers showed that they could have an electronic nature of semiconductors [5]. Later, these suppositions were confirmed experimentally [6]. Moreover, in [6], it was shown that the conductivity exists as in the native protein, as so in denatured. The energy of activation (band gap) changes only. This is important because proteins are very sensitive to temperature. For example, their thermal denaturation occurs already at temperatures of the order of 60°С to 70°С. It is mortal for the vibrational and configurational degrees of freedom. The weak sensitiveness of the electronic subsystem to the temperature allows us further not to take temperature into account. The article [7], the purpose of which was the experimental ground of tunnel mechanism of the transfer of electrons, also results in indirect proofs of band transfer of electrons. They consist in the periods of such transfer that is anomalous for a tunnel mechanism. Finally, in [8], the injection of an electron in the bottom of the conductivity band has been studied theoretically in the representation of numbers of filling. But all calculations listed above, as was mentioned in [9], were executed without taking into account of fact of amino acid heterogeneity of protein systems. However, this heterogeneity destroys one of the basic elements of coherence, namely, homogeneity. Analysis of the influence of the inhomogeneous environment on the coherence of a protein-like system was carried out in [10] and may also be useful to analyze the effect of the amino acid heterogeneity. The analysis of the possibility of considering the heterogeneity of amino acids as small perturbation in relation to some middle-homogeneous system is executed in the article.

Indeed, the presence of the repeated peptide groups and unique radical groups allows the examination of protein molecules at the level of its primary structure as almost periodic formation of a crystalline type. The presence of unidentical radicals does not influence substantially on the electronic configuration of protein molecules, as radicals do not take part in the formation of the primary structure. But their presence, at the same time, substantially complicates an elementary cell, and this is reflected on the structure of the spectra of the electronic states. As the calculations of the energy electronic structure of the real proteins are bulky enough, some farther analysis will be conducted in order to construct a model maximally close to the real protein molecule.

In the paper [11], Suprun and Shmeleva noted that the protein is a typical double nanostructure. It depends on the examined structural level of the protein. In [11], the protein was examined a secondary structure, at a level in which the protein manifests itself as a nanotube. Here, we consider the primary structure of the protein, which is the nanowire for electronic transport in the metabolism (redox process). It was found that due to the heterogeneity, caused by radicals of amino acids, there appears a long-range residual field along the protein nanowire. Its potential energy was found. The analysis of the average electronic configuration of the molecule of the protein was executed for this purpose. It is shown that at such average analysis, a considerable part of interactions in potential energy of the protein molecule is compensated in the sense of long-range action. But there are two uncompensated factors. One of them is the interaction of electrons with the nuclei of atoms of oxygen, the other - with the nuclei of atoms of hydrogen. Both factors are indeed conditioned by radicals. It is shown that even in the absence of the external field, the effective field is not equal to zero. It provides the active transport of electrons along the primary structure of the protein molecule. The external field creates a locally regulative influence on the electron transport.

## Methods

### Nitrogen-oxygen model

The first, enough quality, attempts of quantum calculation of proteins, as it was already marked in [5], refers yet to the 1940s of the last century. The study examined a periodically repeated structure of a protein molecule-peptide group HCNO. But the peptide group is a very ‘strong’ approximation for proteins.

In this article, the real protein will be analyzed without preliminary simplifications. The structural chemical formula of its primary structure looks so:

1 $$ \begin{array}{c}\hfill \kern0.5em \mathrm{O}\kern0.72em \mathrm{H}\kern5.73em \mathrm{O}\kern0.72em \mathrm{H}\kern3.25em \hfill \\ {}\hfill \kern0.49em \left|\left|\kern0.84em \right|\kern5.99em \right|\left|\kern0.84em \right|\kern3.5em \hfill \\ {}\hfill \kern0.48em \mathrm{H}\mathrm{O}-\mathrm{C}-\mathrm{C}-\dots -\mathrm{N}-\mathrm{C}-\mathrm{C}-\dots -\mathrm{N}-\mathrm{H}\hfill \\ {}\hfill \kern4.25em \left|\kern3.18em \right|\kern2.69em \left|\kern3.05em \right|\kern0.75em \hfill \\ {}\hfill \kern4.66em \mathrm{R}\kern2.81em \mathrm{H}\kern2.86em \mathrm{R}\kern2.81em \mathrm{H}\kern2.25em \hfill \end{array} $$

As seen, it consists of three heterogeneous fragments. The left-end group is a carboxyl group. The right-end group is an amino group. Amino acid residues are represented in the middle. Their radicals are marked by a symbol R. If only all radicals were identical, amino acid residues would have been the periodically repeated molecular groups. At that, case analysis of properties of the base electronic configuration of proteins would be considerably easier. In fact, it would be reduced, with satisfactory accuracy, to the analysis of a separate amino acid residue. But amino acid residues are not identical. There are 19 basic amino acids and one amino acid (proline) from which proteins are built. They differ exactly by radicals. Therefore, finding out the electronic configuration of a protein molecule and the type of its crystallinity requires the certain average analysis.

Determination of the optimal average model of the electron subsystem of the protein is necessary to formulate the Hamiltonian operator. This is relatively simple to do in the coordinate representation. Thus, at once, it is possible to structure it in accordance with a *nitrogen model* in which the charge number is *z* = 7:

2 $$ \begin{array}{c}\hfill \hat{H}={\displaystyle \sum_{i=1}^{N_e}}\left\{\hat{T}\left({\mathbf{r}}_i\right)-{\displaystyle \sum_{n=1}^{N_a}}Q\left(\left|{\mathbf{r}}_i-{\mathbf{R}}_n\right|\right)+\frac{1}{2z}{\displaystyle \sum_{j\left(\ne i\right)}^{N_e}}Q\left(\left|{\mathbf{r}}_i-{\mathbf{r}}_j\right|\right)+\right.\hfill \\ {}\hfill +W\left({\mathbf{r}}_i\right)\left.+\frac{1}{z}{\displaystyle \sum_{n=1}^{N_a}}{\sigma}_nQ\left(\left|{\mathbf{r}}_i-{\mathbf{R}}_n\right|\right)-\frac{1}{z}{\displaystyle \sum_{n=1}^{N_{\mathrm{H}}}}Q\left(\left|{\mathbf{r}}_i-{\mathbf{R}}_{\kern0.36em n}^{\left(\mathrm{H}\right)}\right|\right)\right\}\kern0.24em .\hfill \end{array} $$

Here, *N*_*e*_ is the general number of electrons in a protein molecule. *N*_*a*_ is the total number of heavy atoms, among which carbon (C), nitrogen (N), oxygen (O), and also other atoms included in radicals. *N*_H_ is the total number of atoms of hydrogen. $\hat{T}\left(\mathbf{r}\right)=-\frac{\hslash^2}{2{m}_e}{\varDelta}_{\mathbf{r}}$ is the operator of kinetic energy of an electron, and *r*_*i*_ is the coordinate of every separate electron. $Q(r)\equiv \frac{z{e}^2}{r}$ is the absolute value of energy of Coulomb interaction of electron (charge *e*) with a nitrogen nucleus (charge *ze*). **R**_*n*_ is the coordinate of any of the heavy atoms. *W*(*r*) is the potential energy of interaction of an electron with the external field. The coordinate ${\mathbf{R}}_{\kern0.36em n}^{\left(\mathrm{H}\right)}$ ‘selects’ in a sum by *n* only those summands, which belong to the real atoms of hydrogen. A parameter *σ*_*n*_ takes on a value from the variety {0, ±1}. It depends on that which the heavy atoms belongs to a summand with number *n*. Clearly, that in the examined nitrogen model, the value *σ*_*n*_ = 0 corresponds to the atom of nitrogen, the value *σ*_*n*_ = +1 corresponds to the atom of carbon, and value *σ*_*n*_ = −1 corresponds to the atom of oxygen.

Expression 2 is structured so that its first line actually describes a crystal-like structure of atoms, the nuclei of which are the nuclei of nitrogen. The second line actually contains all the perturbing factors. Namely, the external field *W*(*r*) and two hydrogen-like factors:

$$ \frac{1}{z}{\displaystyle \sum_{n=1}^{N_a}}{\sigma}_nQ\left(\left|{\mathbf{r}}_i-{\mathbf{R}}_n\right|\right)\kern0.24em \mathrm{and}\kern0.24em \frac{1}{z}{\displaystyle \sum_{n=1}^{N_{\mathrm{H}}}}Q\left(\left|{\mathbf{r}}_i-{\mathbf{R}}_{\kern0.36em n}^{\left(\mathrm{H}\right)}\right|\right) $$

Such structuring is necessary for the subsequent conversion of the operator (Equation 2) in the numbers of filling representation.

But a nitrogen model belongs only to the average nuclear subsystem. Therefore, it is further needed to do similar estimates for an electronic subsystem. The main contribution to the energy spectrum of electrons gives the heavy atoms (C, N, O). Therefore, for finding out a possible electronic configuration and possible type of crystal, further estimation will be done of the middle number of electrons on one heavy atom (*N*_*e*_/*N*_*a*_).

At first, it is expedient to consider the idealized situation which corresponds to the protein with identical amino acid residues (with the identical radicals R). By a structural chemical formula (Equation 1), it is not difficult to count up the common number of electrons in a protein consisting of *v* with such residues: *N*_*e*_ = (29 + *n*_*e*_)*v* + 10. Here, *n*_*e*_ is the number of electrons in a separate radical R. Denoting, by analogy with electrons, the number of heavy atoms in the separate radical R through *n*_*a*_, we will get:

3 $$ {N}_a=\left(4+{n}_a\right)v+1 $$

The detailed analysis allows to set the relation most optimum from point of electroneutrality of all system:

$$ \frac{N_e}{N_a}=8+\frac{\left({n}_e-8{n}_a-3\right)v+2}{\left(4+{n}_a\right)v+1}. $$

When taking into account the limiting transition *v* → ∞, typical for most proteins (for which *v* > 100; for example, lysozyme consists of 129 amino acids, and bacteriorhodopsin in the membranes of purple bacteria has 248 amino acid residues), the last formula takes the form:

$$ \frac{N_e}{N_a}=8+\frac{n_e-8{n}_a-3}{n_a+4}. $$

Such estimation enables to speak about the *oxygen model* of the electronic configuration of a molecule of protein. And all examined models can be determined as nitrogen-oxygen.

But proteins that would consist of any one amino acid does (do) not happen. In some approximation (zero), we can assume that in all proteins, amino acids are represented equally. Then, it is possible to fulfill the analysis of deviations:

$$ \left(\frac{N_e}{N_a}\right)=\frac{n_e-8{n}_a-3}{n_a+4}, $$

from the point of view of subsequent averaging by amino acid composition. Foremost, the data, needed for such analysis, will be considered. These data are presented in Table 1.

**Table 1:** **Electronic-atomic structure of amino acid radicals**

**Number**	**Amino acid**	***n***_***e***_	***n***_***a***_	*Δ* **(** ***N***_***e***_ **/** ***N***_***a***_ **)**	**Chemical formula of radical**
1	Glycine	1	0	−1/2	-H
2	Alanine	9	1	−2/5	-CH_3_
3	Valine	17	2	−1/3	-CH-2(CH_3_)
4	Leucine	33	4	−1/4	-CH_2_-CH-CH_3_-CH_3_
5	Isoleucine	33	4	−1/4	-CH-CH_3_-CH_2_-CH_3_
6	Phenylalanine	49	7	−10/11	-CH_2_-
7	Tryptophan*	69	10	−1	-CH_2_-
8	Serine*	17	2	−1/3	-CH_2_-OH
9	Threonine*	25	3	−2/7	-CH-CH_3_-OH
10	Methionine	41	4	+3/4	-CH_2_-CH_2_-S-CH_3_
11	Cysteine*	25	2	+1	-CH_2_-S-H
12	Glutamine*	39	5	−4/9	-CH_2_-CH_2_-CO-NH_2_
13	Asparagine*	31	4	−1/2	- CH_2_-CO-NH_2_
14	Aspartic acid (−)	31	4	−1/2	-CH_2_-COOH (−COO^−^)
15	Glutamic acid (−)	39	5	−4/9	−2(CH_2_)-COOH(−COO^−^)
16	Tyrosine (−)	57	8	−5/6	-CH_2_--OH (−O^−^)
17	Histidine (+)	44	6	−7/10	+ -CH_2_-
18	Lysine (+)	41	5	−2/9	−4(CH_2_)-NH_2_ (−NH_3_^+^)
19	Arginine (+)	57	7	−2/11	−3(CH_3_)-NH-CNH-NH (−NH_2_^+^)

Notes of Table 1: The asterisks denote the polar radicals. The ‘+’ or ‘−’ corresponds to the type of charge on the radical and approximate place of its location. Hexagons had shown benzene rings, and pentagons had shown groups of atoms, which have the following composition. In the case of tryptophan, the lower left vertex is a carbon atom, the left-side vertex is a group of CH, the upper vertex has a group of NH, and two common to both rings, the vertices have only carbon atoms. All other vertices, as it should be for benzene, contain groups of CH. In case of histidine, the imidazole ring, starting from the bottom-left vertex counterclockwise, has groups: C, NH, CH, NH, CH.

This table at once demonstrates an interesting anomaly. All except two of the deviations: *Δ*(*N*_*e*_/*N*_*a*_),methionine, and cysteine are negative. Herewith, majority of them, by modulo, is substantially less than 1. Only three have negative deviations close to −1 (phenylalanine, tyrosine, histidine) and one deviation exactly equal to −1 (tryptophan). These four amino acids differ from other ‘negative’ amino acids by having massive molecular rings in their structures. Actually, this explains the large absolute values of deviations.

On another side, methionine and cysteine not only have abnormal signs of deviations *Δ*(*N*_*e*_/*N*_*a*_) but also have abnormally big values: respectively, +3/4 and 1. Cysteine is of particular importance in proteins. It is the ‘fixator’ of the tertiary structure. Therefore, we can assume that all other amino acids with a negative deviation *Δ*(*N*_*e*_/*N*_*a*_) compensate the excess of positive charges of cysteine. Methionine obviously performs an auxiliary role when needed to compensate the negative deviation, but not necessary to fix the tertiary structure.

If now find the average value of the 17 negative deviations *Δ*(*N*_*e*_/*N*_*a*_), it will be about −0.5 of an electron on one heavy atom (in fact, −0.476). On the other hand, a similar mean value of two positive deviations *Δ*(*N*_*e*_/*N*_*a*_) is approximately +1 (in fact, +0.875). This means that if the fragment of protein molecule consisted of *v* = 35 different amino acids, of which one amino acid has average deviation *Δ*(*N*_*e*_/*N*_*a*_) > 0, and the other 34 amino acids (twice by 17) has *Δ*(*N*_*e*_/*N*_*a*_) < 0, the entire fragment would have the general level of deviation approximately equal to zero. And the fragment itself would have an average oxygen-like electron configuration. It is interesting to note that the number of amino acids, 35, on a separate structural fragment of the protein molecule does occur in proteins. For example, such a protein as bacteriorhodopsin (in membranes of purple bacteria) has seven *α*-helical, almost equal, fragments. Each of these fragments contains approximately 35 amino acids (35±1). These facts indirectly confirm the proximity of the electronic configuration of proteins to oxygen.

But there is the charge disbalance related to oxygen electronic configuration. It is conditioned that in a nitrogen-oxygen model, all heavy atoms on the average are examined as nitrogen atoms in the zero approximation, which is represented by the first line in an operator (Equation 2), i.e., have a charge equal to seven, instead of eight, as it is needed for the electroneutrality of all systems. Deviations from this average were handed down in perturbation, and they have the form of summand: $\frac{1}{7}{\displaystyle {\sum}_{n=1}^{N_a}}{\sigma}_nQ\left(\left|{\mathbf{r}}_i-{\mathbf{R}}_n\right|\right)$. Taking this into account, it is clear that the role of ‘compensators’ of excess negative charge can fulfill only the protons of atoms of hydrogen, especially as ‘hydrogen’ electrons are already present in general electron balance that is taken into account by summand:

$$ \frac{1}{z}{\displaystyle {\sum}_{n=1}^{N_{\mathrm{H}}}}Q\left(\left|{\mathbf{r}}_i-{\mathbf{R}}_{\kern0.36em n}^{\left(\mathrm{H}\right)}\right|\right) $$

in an operator (Equation 2). This is obvious also that their average attitude toward heavy atoms must be near 1. We explore this question more in detail, analogous to that as it was done for electrons.

It is easy to estimate from a chemical formula (Equation 1) that for the periodically repeated amino acid residues and one of the final amino group (carboxyl), the number of atoms of hydrogen (protons) is the same and equal (2 + *n*_*H*_). In the other final amino group, this number always equal to 2. Here, by analogy with *n*_*e*_ and *n*_*a*_, the number of protons in a separate radical is denoted by *n*_*H*_. It is not further difficult to define that in all protein molecules (with identical radicals), the complete number of atoms of hydrogen is determined by the formula: *N*_*H*_ = (2 + *n*_*H*_)*v* + 2. Taking Equation 3 into account, after some transformations, it is possible to get:

$$ \frac{N_{\mathrm{H}}}{N_a}=1+\frac{\left({n}_{\mathrm{H}}-{n}_a-2\right)v+1}{\left({n}_a+4\right)v+1}, $$

and in approximation *v* → ∞, we have finally:

$$ \frac{N_{\mathrm{H}}}{N_a}=1+\frac{n_{\mathrm{H}}-{n}_a-2}{n_a+4}. $$

As well as before, the deviation:

$$ \varDelta \left(\frac{N_{\mathrm{H}}}{N_a}\right)=\frac{n_{\mathrm{H}}-{n}_a-2}{n_a+4}, $$

will be explored. The values of these deviations are presented in Table 2. From the table, it is visible that no deviations have sign anomalies. Positive and negative deviations represented approximates equally. Thus, modulo, they are substantially less 1. Two deviations even are exactly equal to 0. Averaging of this deviation, with saving of signs, gives a mean deviation from unit for the relation $\frac{N_{\mathrm{H}}}{N_a}$ only −4.3%. Consequently, with such exactness, it is possible to consider the executed equality *N*_*H*_ = *N*_*a*_ and a protein molecule, accordingly, as electroneutral.

**Table 2:** **Protonic structure of amino acid radicals**

**Number**	**Amino acid**	***n***_***H***_	***n***_***a***_	*Δ* **(** ***N***_**H**_ **/** ***N***_***a***_ **)**	**Chemical formula of radical**
1	Glycine	1	0	−1/4	-H
2	Alanine	3	1	0	-CH_3_
3	Valine	5	2	+1/6	-CH-2(CH_3_)
4	Leucine	9	4	+3/8	-CH_2_-CH-CH_3_-CH_3_
5	Isoleucine	9	4	+3/8	-CH-CH_3_-CH_2_-CH_3_
6	Phenylalanine	7	7	−2/11	-CH_2_-
7	Tryptophan*	8	10	−2/7	-CH_2_-
8	Serine*	3	2	−1/6	-CH_2_-OH
9	Threonine*	5	3	0	-CH-CH_3_-OH
10	Methionine	7	4	+1/8	-CH_2_-CH_2_-S-CH_3_
11	Cysteine*	3	2	−1/6	-CH_2_-S-H
12	Glutamine*	6	5	−1/9	-CH_2_-CH_2_-CO-NH_2_
13	Asparagine*	4	4	−1/4	-CH_2_-CO-NH_2_
14	Aspartic acid (−)	3	4	−3/8	-CH_2_-COOH(−COO^−^)
15	Glutamic acid (−)	5	5	−2/9	−2(CH_2_)-COOH(−COO^−^)
16	Tyrosine (−)	7	8	−1/4	-CH_2_--OH (−O^−^)
17	Histidine (+)	6	6	−1/5	+ -CH_2_-
18	Lysine (+)	10	5	+1/3	−4(CH_2_)-NH_2_(−NH_3_^+^)
19	Arginine (+)	12	7	+3/11	−3(CH_3_)-NH-CNH-NH(−NH_2_^+^)

The notes of Table 2 are the same as the notes of Table 1.

After this analysis, it is possible already to speak about the applicability of description of protein molecules of the nitrogen-oxygen model, the basic feature of which there is the approximate, but enough exact - the error is only in a little percentage, implementation of three inequalities: *z* = 7, *N*_*H*_ = *N*_*a*_, and *N*_*e*_ = 8*N*_*a*_ (or *N*_*e*_ = (*z* + 1)*N*_*a*_). The last of three resulted equalities enables to speak about an energy structure of electronic subsystem of proteins as oxygen-similar.

## Results and discussion

### Analysis of the operator of energy of an electronic subsystem of proteins in a nitrogen-oxygen model: coordinate representation

Now, let us go back to the operator (Equation 2) which will be represented as:

4 $$ \hat{H}={\displaystyle {\sum}_{i=1}^{N_e}}\left(\hat{T}\left({\mathbf{r}}_i\right)-{\displaystyle {\sum}_{n=1}^{N_a}}Q\left(\left|{\mathbf{r}}_i-{\mathbf{R}}_n\right|\right)+\frac{1}{2z}{\displaystyle {\sum}_{j\left(\ne i\right)}^{N_e}}Q\left(\left|{\mathbf{r}}_i-{\mathbf{r}}_j\right|\right)+\tilde{W}\left({\mathbf{r}}_i\right)\right); $$

where it is designated:

5 $$ \tilde{W}\left(\mathbf{r}\right)\equiv W\left(\mathbf{r}\right)+\frac{1}{z}{\displaystyle {\sum}_{n=1}^{N_a}}{\sigma}_nQ\left(\left|\mathbf{r}-{\mathbf{R}}_n\right|\right)-\frac{1}{z}{\displaystyle {\sum}_{n=1}^{N_{\mathrm{H}}}}Q\left(\left|\mathbf{r}-{\mathbf{R}}_{\kern0.24em n}^{\left(\mathrm{H}\right)}\right|\right), $$

and let us analyze the energy of perturbation (Equation 5), taking into account a nitrogen-oxygen model. Energy $\tilde{W}\left(\mathbf{r}\right)$ in the definition (Equation 2) corresponds to the second line. This energy can be interpreted as the effective external field. It consists of the real external field *W*(*r*) and two summands. Exactly these two summands will be analyzed in a sense of the possible existence of residual non-compensated long-range field.

Since a parameter *σ*_*n*_ takes on values 0 and ±1, then the value *σ*_*n*_ = 0, as it was mentioned above, corresponds to the nitrogen atoms. Obviously, that in the corresponding sum of determination (Equation 5), such summands will be absent. Consequently, the effective external field can actually be written as:

6 $$ \tilde{W}\left(\mathbf{r}\right)\equiv W\left(\mathbf{r}\right)+\frac{1}{z}{\displaystyle {\sum}_{n=1}^{N_a}}\left\{Q\left(\left|\mathbf{r}-{\mathbf{R}}_{\kern0.36em n}^{\left(\mathrm{C}\right)}\right|\right)-Q\left(\left|\mathbf{r}-{\mathbf{R}}_{\kern0.36em n}^{\left(\mathrm{O}\right)}\right|\right)\right\}-\frac{1}{z}{\displaystyle {\sum}_{n=1}^{N_{\mathrm{H}}}}Q\left(\left|\mathbf{r}-{\mathbf{R}}_{\kern0.36em n}^{\left(\mathrm{H}\right)}\right|\right), $$

where in the first sum by *n* for radius vectors **R**_*n*_^(*C*)^ and **R**_*n*_^(*O*)^ remains now only ‘living’ summands (by analogy with ${\mathbf{R}}_{\kern0.36em n}^{\left(\mathrm{H}\right)}$ ). The definition (Equation 6) is called the zero-order approximation because it is actually a reformulated precise definition (Equation 5). The numeral values of radius vectors **R**_*n*_^(*C*)^ and **R**_*n*_^(*O*)^ are determined by the concrete examined protein and accepted model of its primary structure.

Now, we will take into account the approximation: *N*_*H*_ = *N*_*a*_. As a result, we will get the insignificant change in relation to previous determination (Equation 6):

7 $$ \tilde{W}\left(\mathbf{r}\right)\cong W\left(\mathbf{r}\right)+\frac{1}{z}{\displaystyle {\sum}_{n=1}^{N_a}}\left\{Q\left(\left|\mathbf{r}-{\mathbf{R}}_{\kern0.36em n}^{\left(\mathrm{C}\right)}\right|\right)-Q\left(\left|\mathbf{r}-{\mathbf{R}}_{\kern0.36em n}^{\left(\mathrm{O}\right)}\right|\right)-Q\left(\left|\mathbf{r}-{\mathbf{R}}_{\kern0.36em n}^{\left(\mathrm{H}\right)}\right|\right)\right\}. $$

About representation (Equation 7), we will speak, as about the first approximation. A chemical formula (Equation 1) and chemical formulas of radicals in Tables 1 and 2 demonstrate that, in most cases, carbon is included in amino acids as the CH_*i*_ groups, *i* = 1, 2, 3, or as the CO groups. Then, for these groups, in some approximation in relation to long-range interaction, it is possible to compensate fully all carbon summands *Q*(|*r* − **R**_*n*_^(*C*)^|) due to two factors. The first is due to the corresponding number of hydrogen summands $Q\left(\left|\mathbf{r}-{\mathbf{R}}_{\kern0.36em n}^{\left(\mathrm{H}\right)}\right|\right)$. At the same time, in the sum over *n* of the basic (unperturbed) part of the Hamiltonian (Equation 4), suitable CH pairs conditionally replaced by nitrogen atoms N. The second is due to the proper amount of oxygen summands. At the same time, in the sum over *n* of the basic (unperturbed) part of the Hamiltonian (Equation 4), suitable CO pairs are conditionally replaced by pairs NN. In such approximation, the examined energy $\tilde{W}\left(\mathbf{r}\right)$ takes the form:

8 $$ \tilde{W}\left(\mathbf{r}\right)\cong W\left(\mathbf{r}\right)-\frac{1}{z}{\displaystyle {\sum}_{n=1}^{N_{\mathrm{O}}}}Q\left(\left|\mathbf{r}-{\mathbf{R}}_{\kern0.24em n}^{\left(\mathrm{O}\right)}\right|\right)-\frac{1}{z}{\displaystyle {\sum}_{n=1}^{N_{\mathrm{H}}^0}}Q\left(\left|\mathbf{r}-{\mathbf{R}}_{\kern0.24em n}^{\left(\mathrm{H}\right)}\right|\right). $$

About the representation (Equation 8) we will speak, as about the second approximation. Here, *N*_*O*_ is number of atoms of oxygen, ‘not compensated’ by a carbon, and *N*_*H*_^0^ is a similar number of atoms of hydrogen. Summands which remained in Equation 8 are related to the OH groups, to the NH_*i*_ groups (*i* = 1, 2), and to the CH_*i*_ groups (*i* = 2, 3). In this sense, they are easily identified in every concrete amino acid, and, consequently, in every concrete protein.

Farther, let us analyze the possibility of comparison of the last two summands at the right part of Equation 8 with the basic potential summand ${\displaystyle {\sum}_{n=1}^{N_a}}Q\left(\left|\mathbf{r}-{\mathbf{R}}_n\right|\right)$ of operator (Equation 4). For this purpose, let us take into account that in a sum ${\displaystyle {\sum}_{n=1}^{N_a}}Q\left(\left|\mathbf{r}-{\mathbf{R}}_n\right|\right)$, there are all *N*_*a*_ summands. At the same time, in the sum ${\displaystyle {\sum}_{n=1}^{N_{\mathrm{O}}}}Q\left(\left|\mathbf{r}-{\mathbf{R}}_{\kern0.24em n}^{\left(\mathrm{O}\right)}\right|\right)$ of expression (Equation 8), the number of summands in *N*_*O*_/*N*_*a*_ times less, and in a sum ${\displaystyle {\sum}_{n=1}^{N_{\mathrm{H}}^0}}Q\left(\left|\mathbf{r}-{\mathbf{R}}_{\kern0.24em n}^{\left(\mathrm{H}\right)}\right|\right)$, this number in *N*_*H*_^0^/*N*_*a*_ times less. Using the chemical formulas of radicals, given in Tables 1 and 2, and chemical formulas of the periodic and terminal molecular groups of protein molecule, given in Equation 1, it is possible to find estimations:

9 $$ \frac{N_{\mathrm{O}}}{N_a}\sim \frac{1}{27},\kern0.5em \frac{N_{\mathrm{H}}^0}{N_a}\sim \frac{1}{2}. $$

Taking into account these estimations, it is possible to get already the third approximation. Consistently, we have:

10 $$ \begin{array}{c}\hfill \tilde{W}\left(\mathbf{r}\right)\cong W\left(\mathbf{r}\right)-\frac{N_{\mathrm{O}}}{z{N}_a}{\displaystyle {\sum}_{n=1}^{N_a}}Q\left(\left|\mathbf{r}-{\mathbf{R}}_n\right|\right)-\frac{N_{\mathrm{H}}^0}{z{N}_a}{\displaystyle {\sum}_{n=1}^{N_a}}Q\left(\left|\mathbf{r}-{\mathbf{R}}_n\right|\right)\equiv \hfill \\ {}\hfill \equiv W\left(\mathbf{r}\right)-\left(\frac{N_{\mathrm{O}}}{z{N}_a}+\frac{N_{\mathrm{H}}^0}{z{N}_a}\right){\displaystyle {\sum}_{n=1}^{N_a}}Q\left(\left|\mathbf{r}-{\mathbf{R}}_n\right|\right)\equiv \hfill \\ {}\hfill \equiv W\left(\mathbf{r}\right)-\left(\frac{1}{189}+\frac{1}{14}\right){\displaystyle {\sum}_{n=1}^{N_a}}Q\left(\left|\mathbf{r}-{\mathbf{R}}_n\right|\right)\cong W\left(\mathbf{r}\right)-\frac{1}{13}{\displaystyle {\sum}_{n=1}^{N_a}}Q\left(\left|\mathbf{r}-{\mathbf{R}}_n\right|\right).\hfill \end{array} $$

Such third approximation has exceptionally quality status. Its main purpose is to demonstrate the presence of uncompensated supplements. This supplement is determined by heterogeneity of the protein and is competing with the external field. This approximation, similarly as the previous two, is important from the practical point of view. It not only confirms the fact of influencing of the external fields on physiology processes, in particular on the ATP synthesis, but also specifies the concrete place of such influence and methods of estimation of its size and direction. In particular, the transfer of charge through the primary structure of protein can be blocked largely, if the external field, directly or in average, satisfies a condition:

$$ W\left(\mathbf{r}\right)\sim \frac{1}{13}{\displaystyle {\sum}_{n=1}^{N_a}}Q\left(\left|\mathbf{r}-{\mathbf{R}}_n\right|\right). $$

If the value of uncompensated field is substantially less, then the obtained physiological value: $\frac{1}{13}{\displaystyle {\sum}_{n=1}^{N_a}}Q\left(\left|\mathbf{r}-{\mathbf{R}}_n\right|\right)$ (due to any violations, for example, illnesses), the external field can resume normal operation of the mechanism of electron transfer through the primary structure of the protein.

Consequently, in an operator (Equation 4), it is possible to use five determinations of the effective external field:

- Exact determination (Equation 5);
- Zero approaching (Equation 6) which is actually a differently formulated exact determination (Equation 5);
- The first approximation (Equation 7) which uses approximate equality *N*_*H*_ = *N*_*a*_;
- The second approximation (Equation 8) which already is substantial, as in it is used as a possibility of formal replacement of pairs CH and CO in the basic part of operator (Equation 4) (second summand) by nitrogens in a long-range-action area;
- The third approximation (10) which already is so substantial that, presumably, may be used for quality evaluations only because residual potential energy in it is taken into account in average along all primary structures of the molecule of protein.

But from all resulted approximations, presumably, most interesting is third. It is interesting because clearly demonstrate the presence of the spatially distributed residual electrostatic field conditioned by heterogeneity of amino acid composition of protein molecule. It does not disappear even on condition of absence of the external field (*W*(*r*) ≡ 0) and is negative. It means that an electron as if ‘sucked in’ to a protein molecule, i.e. remaining potential energy operates as ‘electronic pump’.

### Operator of energy of an electronic subsystem of proteins in a nitrogen-oxygen model: a representation of numbers of filling

An operator (Equation 4) is not very comfortable for the subsequent calculations of the electronic states of conductivity and actual current. Instead, it is comfortable to do this in representation of numbers of filling. It is known also as the second quantization representation or field representation. Therefore, in conclusion, we will formulate an operator (Equation 4) in such representation.

The analysis, which was fulfilled above, allows us to define fully a transformation to the representation of numbers of filling namely:

1. Based on the average nitrogen atomic model, to use as a basis for changes into the representation of numbers of filling one-electron wave functions of the nitrogen ion. They are fully determined and have such a property: *φ*_*f***n**_(**r**) ≡ *φ*_*f*_ (**r** − **n**), i.e., centered by a coordinate of the nuclei: **n** ≡ **R**_*n*_. Here, *f* is a standard set of hydrogen-like quantum states, where *f*_0_ = 1, 2, … is the principal quantum number, *f*_1_ = 0, 1, …, *f*_0_ − 1 is the orbital quantum number, *f*_2_ = 0, ± 1, …, ± *f*_1_ is the azimuth quantum number.
2. Based on the average electron configuration of oxygen, to consider as a nanowire of the semiconductor type with four completely filled energy bands (groups of bands).

After the resulted determinations of base for transformation, the Hamiltonian operator of protein molecule in representation of the numbers of filling will look so [12]:

11 $$ \begin{array}{c}\hfill \hat{H}={\displaystyle {\sum}_{f\mathbf{n}}}{\varepsilon}_f{b}_{f\mathbf{n}}^{+}{b}_{f\mathbf{n}}-{\displaystyle {\sum}_{f\mathbf{n}}}{\displaystyle {\sum}_{\mathbf{l}}}{\displaystyle {\sum}_{g\;\mathbf{m}}}{}{}^{//}Q_{\mathbf{n}\;\mathbf{l}\;\mathbf{m}}^{\; fg}\;{b}_{f\mathbf{n}}^{+}{b}_{g\;\mathbf{m}}+\hfill \\ {}\hfill +\frac{1}{2}{\displaystyle {\sum}_{f\mathbf{n}}}{\displaystyle {\sum}_{g\;\mathbf{m}}}{\displaystyle {\sum}_{f^{\hbox{'}}\;{\mathbf{n}}^{\hbox{'}}}}{\displaystyle \sum_{g^{\hbox{'}}\;{\mathbf{m}}^{\hbox{'}}}}{V}_{\kern0.24em \mathbf{nm}{\mathbf{n}}^{\hbox{'}}{\mathbf{m}}^{\hbox{'}}}^{fg\;{f}^{\hbox{'}}\;{g}^{\hbox{'}}}\;{b}_{f\mathbf{n}}^{+}{b}_{g\;\mathbf{m}}^{+}{b}_{f^{\hbox{'}}\;{\mathbf{n}}^{\hbox{'}}}{b}_{g^{\hbox{'}}\;{\mathbf{m}}^{\hbox{'}}}+\hfill \\ {}\hfill \hfill \\ {}\hfill +{\displaystyle {\sum}_{f\mathbf{n}}}{\displaystyle {\sum}_{g\;\mathbf{m}}}{\tilde{W}}_{\mathbf{n}\mathbf{m}}^{f\;g}\;{b}_{f\mathbf{n}}^{+}{b}_{g\;\mathbf{m}},\hfill \end{array} $$

where the double prime on the summation symbol indicates absence of the summands, in which **n** = **l** = **m**. Matrix elements are determined here by correlations:

$$ {\varepsilon}_f=\left\langle {\varphi}_f\left(\mathbf{r}\right)\right|\hat{T}\left(\mathbf{r}\right)-Q(r)\left|{\varphi}_f\left(\mathbf{r}\right)\right.=-{\left(\frac{z}{f_0}\right)}^2\frac{m_e{e}^4}{2\;{\hslash}^2}; $$

*Q*_**nlm**_^*fg*^ ≡ 〈*φ*_*f***n**_(**r**)|*Q*(|**r** − **l**|)|*φ*_*g***m**_(**r**)〉;

$$ {V}_{\kern0.24em \mathbf{nm}{\mathbf{n}}^{\hbox{'}}{\mathbf{m}}^{\hbox{'}}}^{fg\;{f}^{\hbox{'}}\;{g}^{\hbox{'}}}\equiv \frac{1}{z}\left\langle {\varphi}_{f\mathbf{n}}\left({\mathbf{r}}_1\right){\varphi}_{g\;\mathbf{m}}\left({\mathbf{r}}_2\right)\right|Q\left(\left|{\mathbf{r}}_1-{\mathbf{r}}_2\right|\right)\left|{\varphi}_{f^{\hbox{'}}\;{\mathbf{n}}^{\hbox{'}}}\left({\mathbf{r}}_2\right){\varphi}_{g^{\hbox{'}}\;{\mathbf{m}}^{\hbox{'}}}\left({\mathbf{r}}_1\right)\right.; $$

$$ {\tilde{W}}_{\mathbf{nm}}^{f\;g}\equiv \left\langle {\varphi}_{f\mathbf{n}}\left(\mathbf{r}\right)\right|\;\tilde{W}\left(\mathbf{r}\right)\;\left|{\varphi}_{g\;\mathbf{m}}\left(\mathbf{r}\right)\right\rangle . $$

The concrete type of energy $\tilde{W}\left(\mathbf{r}\right)$ depends on the use of one of the approximations determinated in Equations 5, 6, 7, 8 and 10.

Operators of filling and release (creation and annihilation) of the electronic states *b*_*f***n**_^+^, *b*_*f***n**_ satisfies the anticommutation relations:

$$ {b_f}_{\mathbf{n}}{b}_g{{}_{\mathbf{m}}}^{+}+{b}_g{{}_{\mathbf{m}}}^{+}{b_f}_{\mathbf{n}}={\delta}_{fg}{\delta}_{\mathbf{n}\mathbf{m}}; $$

$$ {b_f}_{\mathbf{n}}{b_g}_{\mathbf{m}}+{b_g}_{\mathbf{m}}{b_f}_{\mathbf{n}} = 0;{b}_f{{}_{\mathbf{n}}}^{+}{b}_g{{}_{\mathbf{m}}}^{+}+{b}_g{{}_{\mathbf{m}}}^{+}{b}_f{{}_{\mathbf{n}}}^{+} = 0, $$

and act on the function of numbers of filling |…, *N*_*f***n**_, …〉. In these functions, variable *N*_*f***n**_ takes on only two values: *N*_*f***n**_ = 0, if the state is not filled, and *N*_*f***n**_ = 1, if it is filled. The method of action of operators *b*_*f***n**_^+^, *b*_*f***n**_ on the function of numbers of filling |…, *N*_*f***n**_, …〉 is determined by the following relations:

$$ {b}_{f\mathbf{n}}^{+}\left| \dots, {N}_{f\mathbf{n}},\dots \right\rangle ={\left(-1\right)}^{\chi_{f\mathbf{n}}}\left(1-{N}_{f\mathbf{n}}\right)\left| \dots, 1-{N}_{f\mathbf{n}},\dots \right.; $$

$$ {b}_{f\mathbf{n}}\left| \dots, {N}_{f\mathbf{n}},\dots \right\rangle ={\left(-1\right)}^{\chi_{f\mathbf{n}}}{N}_{f\mathbf{n}}\left| \dots, 1-{N}_{f\mathbf{n}},\dots \right.. $$

A degree *χ*_*f***n**_ is equal to the number of the filled states, which precede to the state *f***n**.

Further, it is necessary to formulate the wave function of the electron, injected into the protein nanowire, finding the corresponding vector of the current state and the states themselves. These results will be discussed subsequently.

## Conclusions

The possibility of application of the representation of the numbers of filling to the protein molecule, as to the nanowire, was analyzed. A problem is in the fact that this representation requires homogeneity of the system, while the protein molecule is a substantial heterogeneous system. For this purpose, the analysis of averaged electron atomic configuration of the protein molecule was fulfilled. It is shown that in the satisfactory enough approximation of the molecule of protein, it is possible to examine in average a semiconductor system, which consists of nitrogen atoms, but has oxygen electronic configuration. It is the foundation for the executed transition into the representation of the numbers of filling.
